# From Gut to Brain: Glyphosate and Triclosan Impair Microbiome Composition, Neuroactive Metabolites, and Cognitive and Ecological Fitness in *Daphnia magna*

**DOI:** 10.1021/acs.est.5c15302

**Published:** 2026-01-05

**Authors:** Irene Romero-Alfano, Alba Julia López, Benjamin Piña, Cristian Gómez-Canela, Carlos Barata

**Affiliations:** † Department of Environmental Chemistry, 203229 Instituto de Diagnostico Ambiental y Estudios Del Agua (IDAEA-CSIC), Jordi Girona 18, Barcelona 08034, Spain; ‡ Department of Analytical and Applied Chemistry, 16522 Universitat Ramon Llull IQS School of Engineering, Via Augusta 390, Barcelona 08017, Spain; § Department of Analytical and Applied Chemistry, 16522 Universitat Ramon Llull IQS School of Engineering, Via Augusta 390, Barcelona 08017, Spain

**Keywords:** glyphosate, triclosan, behavior, microbiome, gut–brain axis, Daphnia

## Abstract

Gut microbiome dysbiosis is a major off-target effect of many pharmaceuticals, personal care products (PPCP), and plant protection products (PPP). This study aims to characterize these effects for two compounds, glyphosate (a PPP) and triclosan (a PPCP), in *Daphnia magna* juveniles and to trace the downstream consequences for gut- and brain-associated metabolite levels, reproductive performance, and behavior. Both compounds altered levels of neurotransmitters and related metabolites in both head and gut at the ppb–ppt dose range, promoting anxiogenic behavior and inhibiting reproductive traits in a concentration-related manner. These effects occurred concomitantly with alterations in the gut microbiome, analyzed by 16S rDNA sequencing. Correlation analyses between the observed metabolic, reproductive, and behavioral effects and the changes in the metabolic pathway prediction for the treated gut microbiomes revealed an enrichment in pathways related to the biosynthesis of vitamins, of essential fatty acids, and production of short chain fatty acids, which are known to affect systemic serotonin levels. The results suggest a direct link between gut microbiome dysbiosis and cognitive and reproduction effects in *D. magna*, with implications for the environmental and human health hazard assessment of these and other substances with broad antimicrobial spectra.

## Graphical abstract

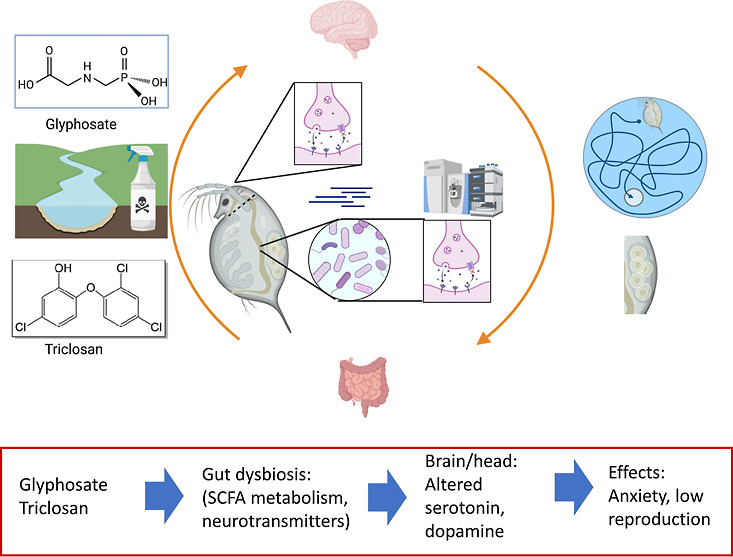

## Introduction

Anthropogenic pollution of freshwater ecosystems by synthetic biocidal and herbicidal compounds has become a pressing concern for ecological health and biodiversity. Among the many stressors, the herbicide glyphosate and the antimicrobial triclosan are ubiquitous contaminants with potential to disrupt non-target organisms.^1,2^ Their effects on aquatic invertebrates, particularly through perturbations of the microbiome, downstream metabolite pathways, and apical responses remain incompletely understood.^3,4^ Such disruptions are able to induce alterations in reproduction and behavior of the freshwater crustacean *Daphnia magna*, a model organism for ecotoxicology and an important keystone species in freshwater food webs.^5,6^

The gut microbiome is now recognized as a crucial modulator of host metabolism, immune function, and nervous system signaling through the so-called gut–brain axis. Metabolites produced by the gut microbiota metabolism, including short-chain fatty acids (SCFAs) like acetate, butyrate, and propionate, serve as key messengers of the microbiota–gut–brain axis (MGBA).^7^ Microbial taxa are involved in the synthesis or modulation of metabolites and precursors that feed into major neurotransmitter systems—including the serotonergic (e.g., via tryptophan → serotonin) pathways,^8,9^ the dopaminergic/catecholaminergic system via microbial modulation of tyrosine or phenylalanine precursors,^10,11^ the GABAergic/glutamatergic balance,^12^ and the cholinergic pathway.^13^ Disruption of the microbiome community structure, or dysbiosis, can alter the production of these neurotransmitters, leading to downstream effects on neural functions, behavior, and reproduction^10,14^

Glyphosate, by targeting the shikimate pathway—which many bacterial groups use to synthesize aromatic amino acids—may reduce microbial production of tryptophan and phenylalanine/tyrosine, precursors to serotonin and the catecholamines.^15^ Studies in zebrafish showed that environmentally relevant glyphosate exposure causes gut dysbiosis, alters central and peripheral serotonin levels, and increases dopamine concentrations in the brain, concomitant with behavioral changes such as elevated anxiety and altered social interactions.^16^

Similarly, triclosan has been shown to interfere with multiple neurotransmitter systems. Chronic exposure of zebrafish to triclosan affects dopamine activity, induces apoptosis of brain neurons, and impairs development and neurobehavioral functions.^17^ In addition, triclosan inhibits acetylcholinesterase (AChE) activity, which interferes with cholinergic signaling; this has been observed both in vitro and in vivo, often associated with oxidative stress or altered gene expression of cholinergic components.^17,18^ Triclosan also appears to disturb the GABAergic system when combined with other pharmaceuticals in zebrafish.^19^

Empirical studies show that glyphosate can impair survival and reproduction across generations in *D. magna*. For instance, exposure to commercial glyphosate formulations and/or mixtures with other compounds (such as atrazine) have been found to reduce the reproductive output, affect maturation, increase oxidative stress markers, and disrupt the gut microbiome in Daphnia.^20,21^ Similarly, triclosan has been shown to reduce the number of neonates per female, decrease body length, lower intrinsic growth rate, and alter antioxidative enzyme activities.^22^ While direct studies in *D. magna* concerning glyphosate or triclosan effects on cholinergic, GABAergic, or catecholaminergic/serotonergic signaling are lacking, there is reported information showing that *D. magna* behavior responds to manipulations of the monoamine systems. For example, modulation of MAO (monoamine oxidase) activity via pharmacological inhibitors in *D. magna* alters serotonin, dopamine levels, and results in measurable changes in locomotor activity and habituation.^23^ There is also evidence that *Daphnia* gut microbiome produces the dopamine precursor l-DOPA and that the gut wall contains several enzymes/genes of the dopaminergic metabolic pathway.^24^

This study aims to elucidate how glyphosate and triclosan pesticide exposure affect the gut microbial community of *D. magna* and to trace the downstream consequences for gut-derived and brain-associated metabolites, reproductive performance, and behavior. By integrating microbiome profiling, metabolomic analyses, and behavioral assays under exposure to environmentally relevant concentrations, we intend to clarify whether perturbations of microbial metabolite production can mediate neurological and life-history effects. Such understanding is essential for risk assessment of chemical pollutants and for re-evaluating regulatory thresholds that account not only for lethality, but also for subtle, sublethal effects which may impair ecosystem functioning.

The herbicide glyphosate is applied to the field as commercial formulations whose surfactants co-formulants have been reported to be more toxic than the active compounds.^25^ Glyphosate co-formulants are being changed continuously to become less toxic, but the toxicity of many of them is still largely unknown.^25^ On the other hand, triclosan is often included as a biocide agent in many personal care products such as toothpaste or soaps.^26^ Thus, triclosan is also consumed, combined with many different products. For this reason, in this study, we worked with the pure ingredients to allow a proper comparison of their bioactivity/effects. The inclusion of two compounds, having different modes of action (herbicide vs antimicrobial) but both possessing bactericide properties, is meant to strength the hypothesis that gut dysbiosis per se explained the neurological related effects on behavior and reproduction in *D. magna*.

## Materials and Methods

### Experimental Animals and Culture Conditions

Parthenogenetic cultures of *D. magna* clone F were used for this study. This clone has been maintained for over 20 years in our laboratory,^27^ and its deep knowledge of molecular and physiological responses has allowed to develop simplified systems for exploring other mammalian type similar effects, such as that of obesogens and antidepressive drugs on metabolic and neuroendocrine disruption.^28,29^ This means that results obtained with this lab clone may help to characterize also a simplified gut–brain mechanism in a non-mammalian species. Animals were cultured under a 16 h light: 8 h dark photoperiod cycle and at 20 ± 1 °C. Several bulk cultures of 10 adult *Daphnia* females were maintained in 2 L of lab water, i.e., ASTM hard synthetic water^30^ complemented with a food additive which is a seaweed extract (added to the water) (Marinure, UK) using a food ratio of 5 × 10^5^ cells/mL of *Chlorella vulgaris* that was cultured in semiaxenic conditions.^27^ Culture media were changed every other day. From third to fifth brood, neonates collected within the first 12 h of being released by their mothers from the adult bulk cultures were used to initiate life-history exposure experiments.

### Life-History Exposure Experiments and Sample Collection

Two independent identical experiments separated by one month were conducted for each of the two tested chemicals (4 experiments in total). In each experiment, new born *D. magna* juveniles (<24 h old) were exposed to five concentrations of glyphosate (99% purity, Chem Services) (10, 100, 1000 ng/L, 10, 100 μg/L) or to four concentrations of triclosan (99%, GC, Sigma-Aldrich) (20, 200 ng/L, 2, 20 μg/L) for 21 days, as depicted in Figure S1 (Supporting Information). Concentrations were selected to include environmental levels and toxicologically relevant responses. Research across many aquatic reservoirs from 2010 to 2023 detected glyphosate concentrations in surface waters up to 258 μg/L, with average concentrations ranging from 0.05 to 0.85 μg/L.^31^ Comprehensive reviews indicated that surface water concentrations of triclosan with known input of raw wastewater ranged from 11–98 ng/L but can reach residue levels as high as 2 μg/L.^32,33^

Stocks solutions of triclosan were dissolved in acetone, which was used at the same concentrations in all treatments, including the control (0.1 mL/L). Glyphosate stock solutions were dissolved in Milli-Q water and freshly prepared each week. Groups of five *D. magna* juveniles were initially exposed to the tested treatments in 300 mL of test medium using the culture conditions outlined in the previous section. Treatments were replicated 7 times. The test medium was changed completely with freshly prepared media once every 48 h. After 6 days of exposure, juveniles of five replicates (25) were used for behavioral assays and sampled in groups of 5 for either gut microbiome (experiment 1) or metabolomic studies (experiment 2). In experiment 1 following exposures and behavioral assays (Figure S1), juveniles in groups of 5 were transferred to 300 mL of clean autoclaved ASTM water during 12 h to remove any nonsymbiotic bacteria,^34^ their guts dissected in iced-cooled Petri dish, pooled in 0.5 mL of lysing buffer, frozen in N2, and storage at −80 °C until DNA processing. In experiment 2 immediately following behavioral assays, the guts and heads of 5 animals were dissected also in a cold Petri dish, polled in an Eppendorf with 0.5 mL of acetonitrile: water 1:1 buffer, frozen in N_2_, and stored at −80 °C until metabolite processing. In both experiments, the remaining 10 juveniles from the 2 remining replicates were cultured individually in 120 mL borosilicate glass bottles filled with 100 mL of exposure media. Animals were monitored daily for survival and offspring production. Measurements of body length were taken from all adults at the end of exposures, as well as from 10 to 20 neonates released in the last clutch. The body lengths were measured from the head to the base of the spine using a Nikon stereoscope microscope (SMZ 150, Nikon, Barcelona, Spain) along with ImageJ software (http://rsb.info.nih.gov/ij/). The intrinsic rate of increase (*r*) was computed iteratively from the Lotka equation (eq 1) using the measured age, specific survival, and fecundity rates

1 $$\sum_{x=0}^{\infty }e^{-rx}l_{x},m_{x}=1$$

where *l*_*x*_ is the proportion of the females surviving to age *x* (days) and *m*_*x*_ is the number of juveniles produced per surviving female between the ages *x* and *x* + 1. The age at birth was set to 0 and l*x* was set to 1 as mortality was negligible and more related to handling than to treatments.

**Figure 1:**
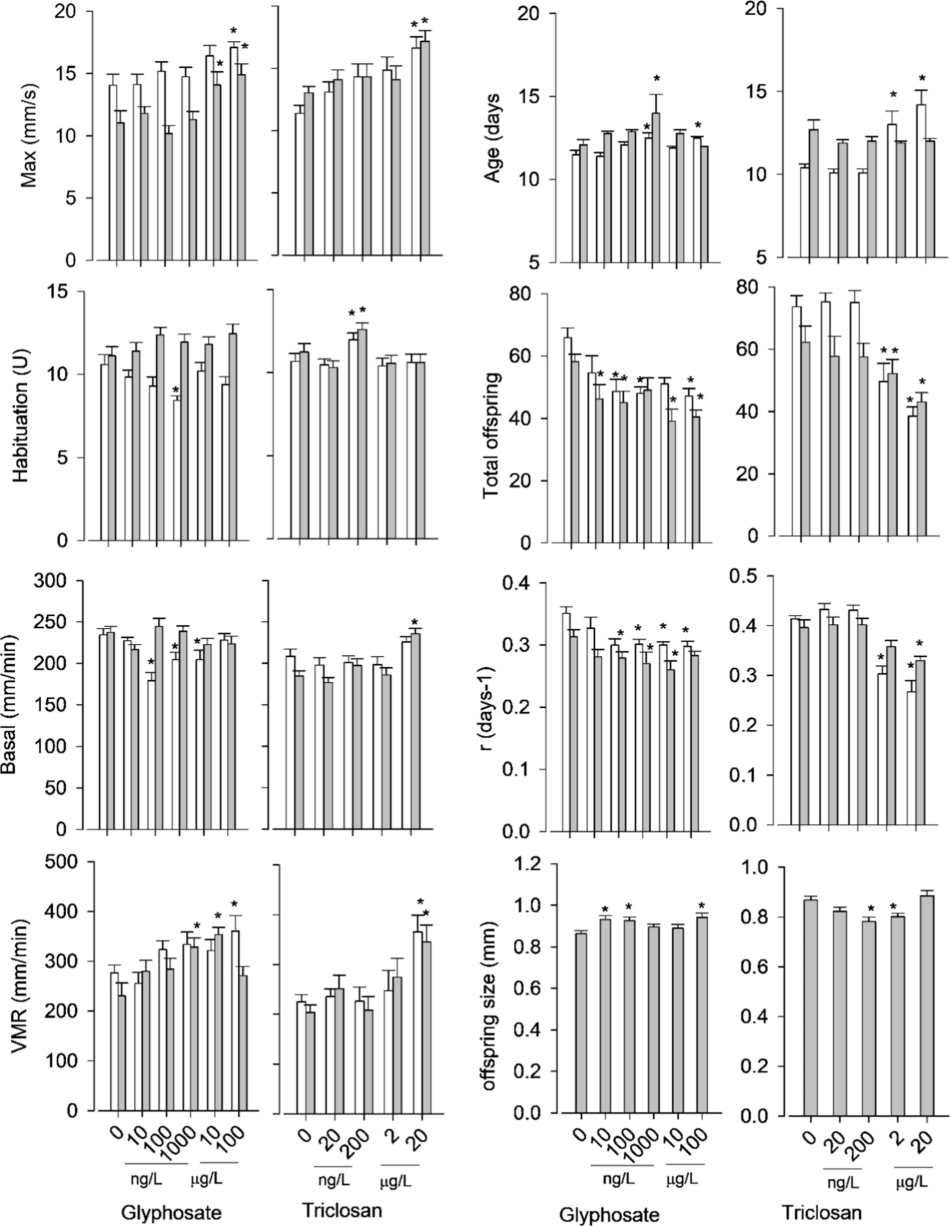
Behavioral (left panel graphs) and reproductive (right panel graphs) *D. magna* responses across the studied treatments and experiments. Values are mean ± SE, *N* = 10–24. * mean significant differences from controls following ANOVA and Dunnetts’s posthoc tests (*p* < 0.05) or the non parametric equivalent ones. White and gray bars correspond to traits from the first and the second experiment.

In experiment 1, 1 L of the tested water samples was collected to analyze their microbiome (Figure S1): water collected from the bottles where *Daphnia* were exposed for 48 h (at the time of medium renewal) and freshly prepared media. Water samples were sequentially filtered through 3 and 0.2 μm membrane filters (Isopore, polycarbonate and Omnipore, PTFE, respectively, Merck-Millipore). Filters were preserved at −20 °C until processed for DNA extraction.

### Chemical Analyses

Accuracy and stability of 10, 100 μg/L glyphosate and 2, 20 μg/L triclosan dilutions were evaluated by duplicate during the exposure period by collecting 10 mL of water samples at 0 (fresh medium), 24 and 48 h in both experiments. Additional physicochemical water quality variables such as pH, conductivity and oxygen levels were also evaluated using a WTW Multi 340i hand-held meter, Labstuff. eu, Godenbergstraße 5, 23,714 Malente, Alemania, and in all cases were within recommended levels: pH-7.5–7.8; conductivity −460–480 μS/cm; oxygen −95–100% saturation levels.

### Behavioral Responses

Two tests were performed to study the effect of glyphosate and triclosan in *D. magna* basal, visual motor (VMR), maximal (Max) and habituation (H) responses to light stimuli, using a DanioVision Observation Chamber (DVOC-0040) and following previous procedures.^23,35,36^ Further information is listed in the Supporting Information.

### DNA Extraction

DNA extraction of gut *D. magna* samples followed the phenol–chloroform method described before^37^ with few modifications. Further details are given in the Supporting Information.

### 16S rRNA Gene Sequencing

Aliquots of each of the DNA samples were sent to Novogene Europe (Cambridge, UK) for high-throughput sequencing. Further details are given in the Supporting Information. Briefly, total DNA preparations of 16S gene fragments of the rRNA gene were amplified using bar-coded universal primers directed to the bacterial V3-V4 16s rRNA region (341F CCTAYGGGRBGCASCAG, 806R GGACTACNNGGGTATCTAAT) (Novogene Europe, UK). All PCR reactions were carried out with Phusion High-Fidelity PCR Master Mix (New England Biolabs). Quality-checked PCR products were mixed at equal density ratios and purified by a Qiagen Gel Extraction Kit (Qiagen, Germany). Sequencing libraries were generated using NEBNext Ultra DNA Library Pre Kit for Illumina, following manufacturer’s recommendations, and index codes were added. Libraries were prepared, and amplicons were sequenced on a paired-end Illumina platform generating 250 bp raw reads, and after reads merging, chimera removal, and DADA2 denoising, ASVs (Amplicon Sequence Variables) were obtained. Annotation of ASVs was done using the SILVA database (v.138) through the Classify-sklearn moduler in QIIME2 software. ASV counts were rarefied using the package vegan (v. 2.6.4, software R, v. 4.2.1) prior to data analysis. Relative abundances were calculated, when necessary, as the abundance of a given taxa divided by the sum of all of them.

### Metabolome Analysis

Neurotransmitters extraction and their related metabolites from both the head and the gut was conducted using previous procedures.^38^ Samples were analyzed using liquid chromatography coupled to a triple quadrupole mass spectrometer (UHPLC-MS/MS) method optimized previously.^39^ Further details are given in the Supporting Information.

### Statistical Analysis and Data Analysis

For each pesticide considered, glyphosate and triclosan, reproductive and behavioral responses across different concentrations and the two experiments performed were compared against controls by two-way ANOVA with concentration and experiment as fixed factors, followed by posthoc Dunnett’s test (*p* < 0.05). Offspring size as well as head and gut metabolites were only measured in one of the experiments so for those responses one way ANOVA was performed. Analyses of *r* were based on Jackknife pseudovalues^40^ obtained using the full data set. Tests were conducted with the aid of the statistical package IBM SPSS v19., and General Linear Models were used instead of conventional ANOVA when more than two factors or unequal replicates across treatments were considered. Prior to analyses, ANOVA assumptions of data normality or variance homoscedasticity were tested and, if necessary, log transformed.^41^ Age at first reproduction was not normally distributed, thus it was analyzed by nonparametric Kruskal–Wallis tests followed by Dunnett’s equivalent nonparametric comparison test.

Analysis of β-diversity was performed using calculated Bray–Curtis distances and represented in a nonmetric multidimensional scaling (NMDS). Significant (*P* < 0.05) differences among microbial communities of *Daphnia*-gut and water microbiomes within and across the two tested substances and the different concentrations was determined using tow experimental designs by the PERmutational Multivariate ANOVA (PERMANOVA) test (vegan package, v. 2.6.4): (1) grouping substance concentrations as high (10, 100 μg/L glyphosate; 2, 20 μg/L triclosan) and low (control, 10, 100, 100 ng/L glyphosate; control, 20, 200 ng/L, triclosan). Since different tested concentrations were used across the tested compounds, the previous design allowed us to compare both compounds across high and low concentrations accounting for both main and interaction effects. (2) Within each compound, glyphosate or triclosan, a further analysis on gut microbiome β-diversity was conducted to account for concentration effects. Microbiome functional predictions based on phylogenetic investigation of communities by reconstruction of unobserved states (PICRUSt) was performed, and MetaCyc pathways abundance were analyzed using Wilcoxon rank-sum test comparing the bacteria abundance of low vs high concentrations for each compound.

For each pesticide, metabolite abundances in the head and gut across pesticide concentrations were compared by nonparametric tests and their relative abundance relative to control treatments was visualized in Heatmaps (MATLAB, v. 24.2.0.2863752; MathWorks, Inc., 2024b). Functional analysis of deregulated metabolites was performed using MetaboAnalysis 6.0.

Spearman’s rank correlation tests followed by hierarchical clustering (ggplot2 package), was performed to relate bacteria relative abundance in the gut with that of metabolites and reproductive and behavioral responses for both tested compounds separately.

## Results

### Glyphosate and Triclosan Stability in Water

The concentrations of glyphosate and triclosan remained consistent in both experiments with minor changes (Table S1, Supporting Information). This indicated that the compounds were stable during the study exposition times.

### Behavioral and Reproduction

Exposure to glyphosate and triclosan altered significantly (*P* < 0.05) most measured behavioral and reproduction related responses across experiments (experiment, treatment, or interaction effects in Table S2, Supporting Information). Glyphosate and triclosan increased Max, VMR, and age at first reproduction and decreased total offspring production and r responses in a concentration related manner (Figure 1). Effects on habituation, basal locomotion activity, and offspring size varied between glyphosate and triclosan treatments. Glyphosate increased habituation (decreased arbitrary units-U values), offspring size, and decreased basal activity at intermediate concentrations at least in one experiment, whereas triclosan did so in the opposite way (Figure 1). Nonsignificant adult body size results are given in Tables S3 and Supporting Information.

## Metabolites

Up to 17 metabolites were detected, and their residue levels quantify in both the head and the gut tissue organs of *D. magna*. Their heatmaps are depicted in Figure 2. Glyphosate significantly altered the residue levels of 14 metabolites in both tissues (Figure 2 A,B), whereas triclosan did so in 7 and 10 of them in the head (Figure 2C) and gut (Figure 2D) tissue, respectively. Interestingly, half of the deregulated metabolites were up and down regulated upon exposure to both compounds. Tyrosine/catecholamine and tryptophan KEGG pathways were highly enriched in both tissues upon exposure to glyphosate and only the former in the gut in daphnids exposed to triclosan (Figure S3).

**Figure 2:**
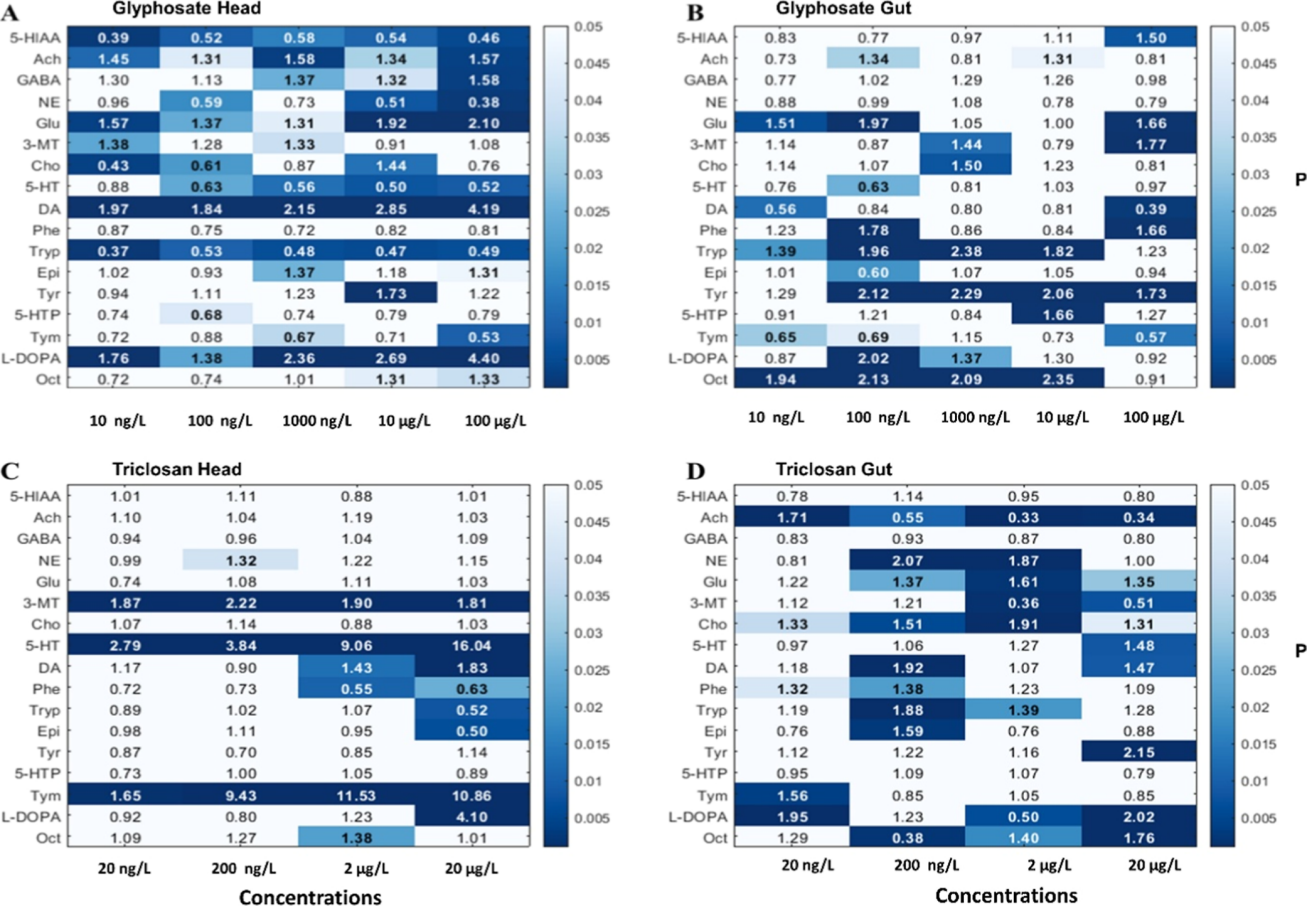
Heatmaps showing metabolomic fold changes relative to the control group. (A,B) Correspond to heat and gut, respectively, following glyphosate exposures, while (C,D) represent heat and gut, respectively, following triclosan exposures. Shading indicates the statistical significance of changes in each analyte, and numbers represent the fold change values. Abbreviations are explained in the Supporting Information.

### Changes in Water and Daphnia-Associated Microbial Communities’ Composition

The number of reads per library ranged from 41,366 to 79,105, with a number of unique ASV per sample from 33 to 145, after chloroplast and mitochondrial sequences. After DADA2 denoising, the percentage of clean reads was an 85% average of raw reads (ranging from 64% to 96%). See Supporting Information file for ASVs, their associated taxonomic groups, their reads across samples and metadata. All sequence data are available at the NCBI SRA under accession number PRJNA1370489.

Notable shifts in taxonomic composition followed glyphosate and triclosan exposures across concentrations for both water and gut microbiome composition, although they were particularly important at high concentrations (10, 100 μg/L glyphosate; 2, 10 μg/L triclosan) (Figure 3A,C). The relative abundance of *Actinobacteriota* and *Patescibacteria* in the *D. magna* gut decreased upon exposure to glyphosate (Figure 3 A), particularly the order *Micrococcales*, while the order *Corynebacteriales* increased. Within the dominant phyla *Proteobacteria* and *Bacteriiodales* (Figure 3A), the relative abundance of *Pseudomonadales* and *Enterobacterales* increased, *Flavobacteriales* decreased, and the Phylum *Burkholderiales* included both increasing and decreasing groups. In water samples, the relative abundances of *Proteobacteria* and *Patescibacteria* increased upon exposure to glyphosate (Figure 3A). Triclosan decreased the relative abundance of *Patescibacteria* (gut and water) and of *Bacteriodonta* (only in water), and increased *Proteobacteria* in water (Figure 3B). The abundance of orders such as *Flavobacteriales* and *Burkholderiales* decreased in both the gut and water upon triclosan exposure, whereas that of *Enterobacterales* *,* *Pseudomonadales*, *Rhodobacterales*, and *Rhizobiales* increased. Differences among the two products (glyphosate vs triclosan), followed by those from low and high concentrations, and their interaction in both gut and water, explained a significant (*P* < 0.05) portion of the variation (*R* ^2^) in bacterial communities’ compositions or β diversity (design 1, PERMANOVA, *P* < 0.05, Table S4, Supporting Information). Within each compound (glyphosate and triclosan), concentration also accounted for a significant portion of the variation (*R* ^2^) in bacterial communities’ composition (see design 2 results PERMANOVA, of microbiome populations *P* < 0.05, Table S4, Supporting Information). The effect on taxonomic composition of both glyphosate and triclosan treatments was revealed by nonmetric multidimensional scaling (NMDS), for both gut and water microbiomes (Figure 3C,D). The functional analysis of the affected bacterial groups at the high studied exposure levels (10, 100 μg/L glyphosate; 2, 10 μg/L triclosan) resolved in 86 enriched MetaCyc bacteria metabolic routes (Table S5, Supporting Information), 46 of them shared by both pesticides and 28 and 14 being unique for triclosan and glyphosate, respectively. Both compounds shared two overrepresented (acetyl-CoA fermentation to butanoate and *Bifidobacterium* shunt) and two underrepresented pathways (mixed acid fermentation and acetylene degradation) involved in the production of short chain fatty acids (SCFA). Glyphosate-treated microbiomes showed two additional underrepresented SCFA producing routes (S)-propane-1,2-diol, l-glutamate degradation VIII (to propanoate), plus the shikimate one (all-chorismate) (Figure 3E). Underrepresented metabolic routes by both pesticides also included several vitamin and fatty acid biosynthesis pathways and the *trans*, octa-*cis* decaprenyl phosphate biosynthesis implicated in the synthesis of a juvenile hormone precursor (farnesyl diphosphate).

**Figure 3:**
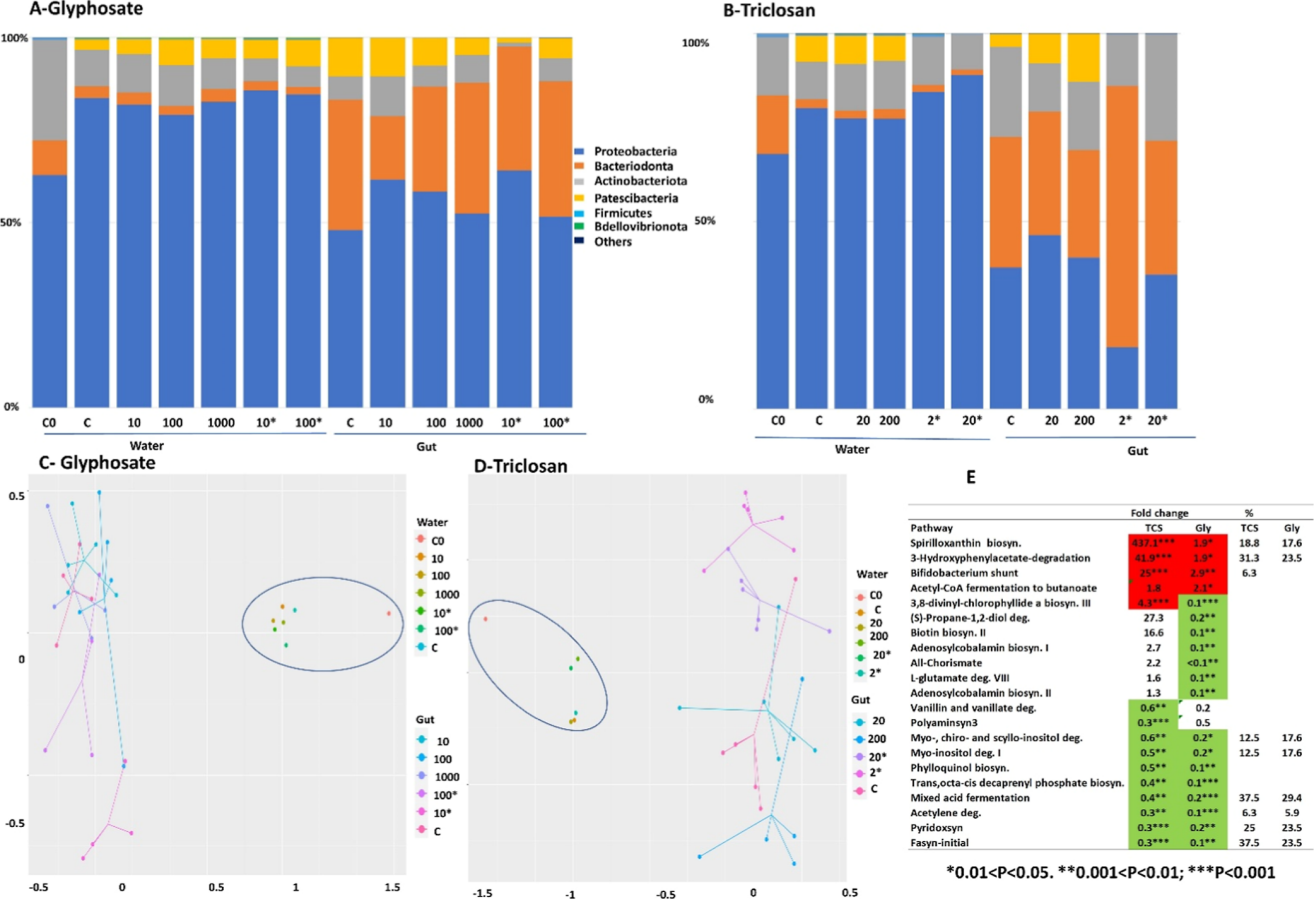
Community composition of microbial communities in *Daphnia*’s gut and water altered following glyphosate (A,C) and triclosan (B,D) exposures. The top Phyla relative abundance was plotted by the different treatments and the other reads grouped as “Other taxa” (graphs A,B). Nonmetric multidimensional scaling (NMDS) based on Bray–Curtis distance matrices are shown in graphs (C,D). In NMDS graphs circles grouped water samples. In legends and *x* axis concentrations are given in ng/L except those with * that are given in μg/L; water bacteria communities in freshly prepared media are depicted as C0. Fold change abundances at high exposure levels vs lower ones of selected predicted pathways that differ significantly between treatments are displayed in a heatmap (E). Graph E also include the % of bacteria whose abundance in glyphosate and tricosan treatments were related with behavioral, reproduction, and metabolome responses in Figure 4. Gly–glyphosate; TCS-triclosan.

### Gut Microbiome vs Metabolite, Behavioral, and Reproductive End Points

The correlations between changes in gut microbiome composition upon glyphosate and triclosan treatment (tested separately) and the concomitant alterations in metabolite, behavioral and reproductive end points were analyzed using non parametric Kendal’s correlation analysis (Figure 4, see also Figures S4 and S5 in Supporting Information). Up to 26 and 30 different bacteria taxa correlated with neurologically related metabolites and behavioral and reproductive traits upon glyphosate and triclosan treatments, respectively (Figure 4 A,B). Hierarchical clustering revealed two major groups of bacteria for both treatments (Figure 4). The larger glyphosate cluster 1 included 17 bacterial groups whose relative abundance positively correlated with the level of head and gut catecholaminergic, GABAergic metabolites, head cholinergic, and gut serotonergic ones, as well as with most behavioral traits, while showing negative correlations with head serotonin, NE, gut NE, offspring production, and fitness (Figure 4A, see also Figure S4, Supporting Information). The glyphosate smaller cluster 2 included 9 taxa that had the opposite correlation patterns as cluster 1, mostly related by the fact that included bacteria having high abundance in control and in the low glyphosate treatment group. Triclosan larger cluster 1 included 16 bacteria whose abundance were positively correlated with most behavioral responses and with levels of serotonergic, dopaminergic/catecholinergic, and cholinergic metabolites in both the head and gut and with GABAergic metabolites in the head (Figure 4B, see also Figure S5, Supporting Information). Bacterial groups in this cluster showed negative correlations with most reproductive traits and head and with gut metabolic precursors and degradation products of the studied neurotransmitter signaling pathways (Figure 4B, see also Figure S5, Supporting Information). Triclosan smaller cluster 2 included 9 taxa whose abundance was greater in control treatments and hence they had the opposite pattern as cluster 1. Interestingly, bacteria integrating cluster 1 for both compounds were associated by PICRUSt with functional metabolic routes involved in the production of short chain fatty acids, energy throughout fermentation, and other catabolic routes involved in the breakdown of lignin and the synthesis of essential vitamins and fatty acids (Figure 3E and Table S5, Supporting Information).

**Figure 4:**
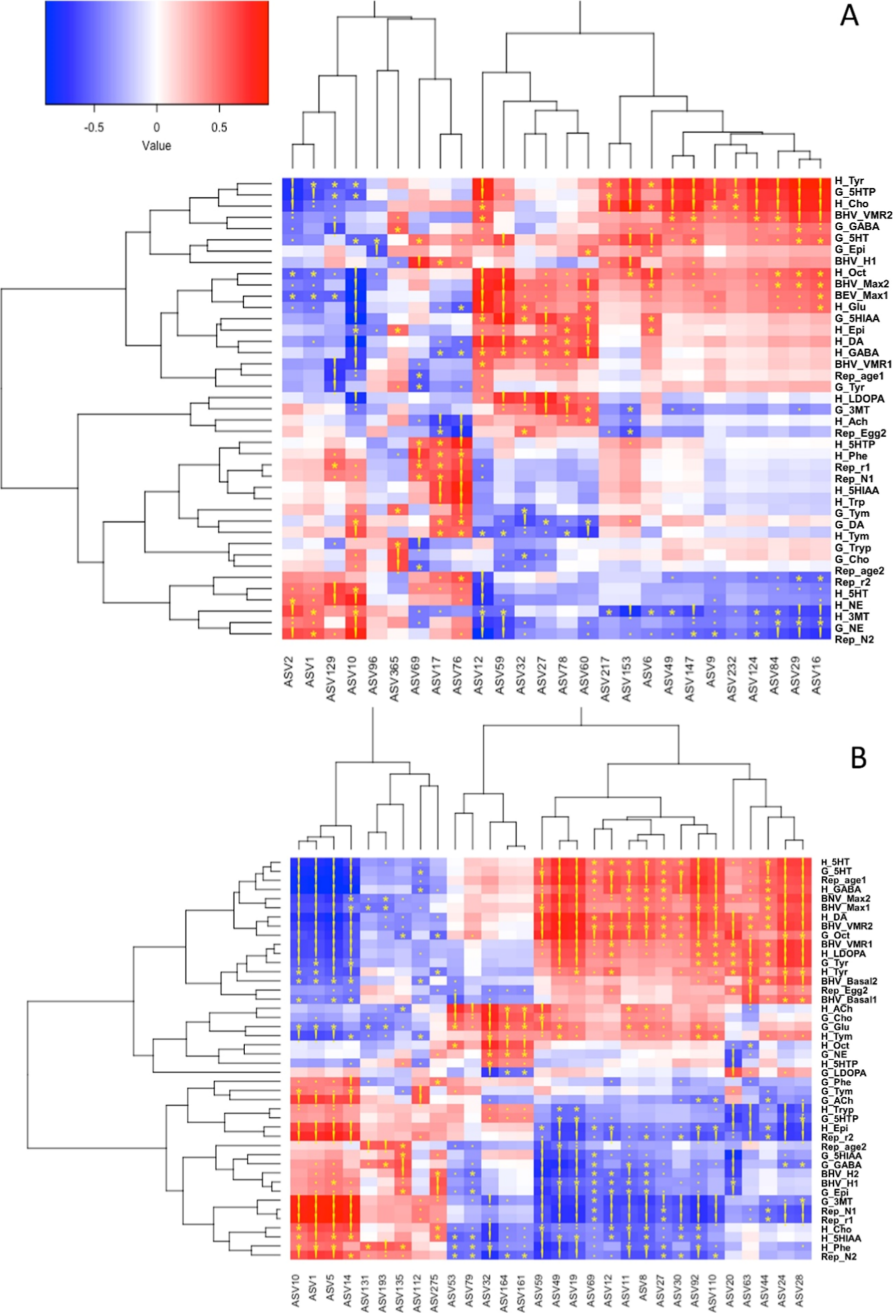
Hierarchical clustering of correlation between gut bacteria, behavioral, reproduction, metabolomic responses of *D. magna* individuals exposed to glyphosate (A) and triclosan (B). Red and blue are positive and negative correlation values. Symbols: “.”, “*” and “!” are 0.05 < *P* < 0.01; 0.01 < *P* < 0.001; *P* < 0.001, respectively. H, G, BHV, and Rep indicate head and gut metabolites, behavioral, and reproduction responses, respectively. The rest of the abbreviations are explained in the Supporting Information.

## Discussion

The two studied compounds proved to be able to enhance the locomotion responses to light (Max and VMR) at relatively high concentrations. Glyphosate increased offspring size and decreased reproduction at 10 and 100 ng/L, respectively, whereas triclosan decreased offspring size at 200 ng/L and 2 μg/L and impaired offspring production at high concentrations (≥2 μg/L). Reproducing *D. magna* females adjust their per offspring and total reproductive investment to maximize offspring fitness across food environments:^42,43^ production of less and larger offspring is favored under limiting food conditions, and the opposite is true when there is plenty of food. Recent work showed that brain serotonin levels are involved in the above-mentioned food-mediated reproductive investment.^29^ Indeed, pharmacological studies demonstrated that selective serotonin reuptake inhibitors (SSRIs) induced the production of more but smaller offspring under food limitation by increasing brain serotonin levels in reproductive females, whereas the opposite reproductive behavior was observed in genetically modified, tryptophan hydroxylase-knockout females lacking serotonin.^29,44^ This means that triclosan, by increasing serotonin levels in the head, decreased the offspring size while keeping offspring production similar to those of controls. Conversely, glyphosate decreased serotonin head levels, hence increased offspring size, and decreased offspring production. It has also been reported that brain serotonin levels ameliorate antipredator scape like responses to light in *Daphnia*, like enhanced VMR and Max responses, whereas dopamine increased them.^23,45^ Increasing VMR and Max responses can also be considered an anxiogenic behavior in *D. magna*.^46^ Both pesticides increased dopamine levels in the head but triclosan also did so for serotonin, which means that VMR and Max responses should be less pronounced upon the triclosan exposure. Therefore, the behavioral results are consistent with the effects on reproduction and agree with the previous argument.

Both pesticides caused gut dysbiosis, in particular, at high exposure levels. Gut dysbiosis has also been reported for glyphosate in *D. magna* ^3^ but not for triclosan. Our data show notable enrichments of specific taxa in *Daphnia*’s gut microbiome, such as an increase in *Burkholderiales*, *Enterobacterales*, and *Pseudomonadales*, particularly for *Aeromonas*, *Pseudomonas,* and *Acidovorax* orders. *Aeromonas* species are pathogenic bacteria widely present in aquatic environments, causing diseases in both humans and aquatic animals.^47,48^ However, some species of *Aeromonas* have also been linked to improved nutrient assimilation and host fitness in *Daphnia*, potentially improving the metabolic support when *Daphnia* is exposed to environmental stressors.^49^ Previous studies have identified significant relationships between *Pseudomonas* and poor dietary conditions in *D. magna*.^37^ *Aeromonas* and/or *Pseudomonas* enrichment in *Daphnia*’s gut also has been associated with exposure to the antibiotic oxytetracycline, the pesticide deltamethrin, microplastics, and mercury.^50–53^ In addition, it has been reported that *Pseudomonas* groups may support *Daphnia*’s adaptation to environmental stressors enhancing the tolerance to mercury and antibiotics,^53,54^ respectively. *Acidovorax* appears across multiple *Daphnia* studies and is often implicated when hosts are shifted by diet, temperature, or cyanobacterial exposure.^37^

Bacterial community alterations were also observed by the decrease of key bacteria of *Daphnia*’s gut microbiome after both treatments, such as *Limnohabitans* and *Flavobacterium*, which have been associated with healthy *D. magna* reproductive individuals.^55,56^ Other dominant gut taxa (*Acidovorax*, *Gemmobacter*, and *Rhizobium*), which are considered key bacteria in the *Daphnia* gut microbiome,^47,57–60^ were also more abundant in control and low concentration treatments.

The strong downregulation of the shikimate pathway by glyphosate is consistent with its bactericide and herbicide mode of action.^61^ Nevertheless, inhibition of the shikimate pathway by glyphosate did not resolve in significant downregulated changes of associated biosynthesis pathways such as of the gut aromatic amino acids tyrosine and tryptophan.^62^ This is likely explained by the fact that *Daphnia* is a phytoplankton grazer and that in the present study grazed on the microalgae *Chlorella*, which is rich in those and other amino acids.^63^ Glyphosate is moderately toxic to *Daphnia* and *Chlorella*, impairing their survival or growth, respectively, at concentrations from one to 3 orders of magnitude greater than the tested in the present study (EC50 = 2–7, 450 mg/L, respectively).^64,65^ This means that tyrosine and tryptophan levels detected in the gut of glyphosate-treated animals were probably coming from algae digestion and amino acid gut absorption.

Many of the enriched MetaCyc pathways of gut microbiomes depicted in Table S5 include amino acid, carbohydrate metabolic routes, and vitamin biosynthesis, which are present in healthy *D. magna* animals.^57^ Up to six and 19 catabolic routes were deregulated exclusively by glyphosate or triclosan, respectively. Glyphosate also reduced the abundance of anabolic routes implicated in the synthesis of vitamins and antioxidant products such as thiol groups as well as the pathways implicated in (S)-propane-1,2-diol degradation and l-glutamate degradation VIII, which produces propanoate and acetate, respectively. In contrast, triclosan increased the representation of pathways implicated in the *Bifidobacterium* shunt and the acetyl-CoA fermentation to butanoate, which produce the SCFAs butyrate and propionate, respectively.^66,67^ Two additional routes also involved in acetate production (mixed acid fermentation and acetylene degradation, https://www.metacyc.org) were underrepresented in triclosan-treated microbiomes. The overall result of these changes is that glyphosate inhibited metabolic production to a greater extent than triclosan SCFA metabolic production. According to the gut microbiota–brain axis communication model,^68^ metabolites produced by the gut microbiota such as short-chain fatty acids (SCFAs) may serve as key messengers of the microbiota–gut–brain axis (MGBA).^7^ SCFAs can cross the blood–brain barrier directly, modulating neurotrophic factor levels to impact brain function, mood, and learning. Furthermore, SCFAs may influence the production of neurotransmitters such as serotonin, which plays a critical role in regulating various brain functions.^7^ This means that the changes in SCFA-producing gut bacterial groups observed in glyphosate-treated animals may explain the observed disruption of serotonin levels in the *D. magna* head. Furthermore, neurotransmitters such as gamma-aminobutyric acid (GABA), serotonin, dopamine, and acetylcholine can also be produced directly by the gut microbiome.^69,70^ Interestingly, dopamine levels decreased, and those of its metabolites 3MT increased in the gut of *D. magna* females exposed to glyphosate. Triclosan affected to a greater extent the neurotransmitters of the gut, decreasing acetylcholine and 3MT, but increasing dopamine, its precursor l-DOPA, octopamine, and serotonin. There is evidence that dopaminergic metabolites can be produced in the gut microbiome of *D. magna* and that the gut tissue contains dopaminergic metabolizing enzymes.^24^ Therefore, the results presented for gut microbiome dysbiosis and deregulated neurotransmitters and related metabolic pathways upon exposure to both pesticides are consistent with the gut microbiome–brain axis communication premise.

Both compounds also reduced the representation in the gut microbiome of the trans, octa-*cis* decaprenyl phosphate biosynthesis pathway, which produces farnesyl diphosphate, the precursor of the *Daphnia* juvenile hormone^71^ that, together with ecdysone, regulate growth and reproduction.^72–74^ Many gut microbiome pathways involved in limiting essential nutrients such as vitamin and fatty acid biosynthesis were also underrepresented in the treated gut microbiomes, which could have also affected reproductive traits.

There was clear evidence that both compounds altered *D magna* gut microbiome, the levels of gut and head neurotransmitters, and reproduction and behavioral responses. Correlation following by hierarchical analysis helped to identify gut bacteria groups and functions whose representation in the microbiome coincides with the extend of these adverse effects. Particularly important is the depletion in pathways related with the SCFA production and fatty acid and vitamin biosynthesis, which provides a direct link between gut microbiome dysbiosis and cognitive and reproduction defects in *D. magna*. This means that the here reported results together with previous ones^5^ provide solid evidence for considering the *D. magna* model a simplified system for exploring similar gut–brain mechanisms in mammals. Nevertheless, two aspects must be further addressed. It is well-known that there is an inherent genetic variability in susceptibility to diseases related with gut dysbiosis across human populations.^75^ *D. magna* gut microbiomes share similar beneficial bacteria but are adapted to local environmental conditions and also vary to some extent across genotypes.^76^ Experimentally microbiome manipulations have shown that the microbiome composition is key in determining *D. magna* adaptation and tolerance to local conditions and stressors.^77^ This means that the *D. magna* model offers a unique possibility to conduct further research with additional clones and microbiomes to account for genetic/population variability in susceptibility for gut dysbiosis. Despite that *D. magna* has a simple but well-organized nervous system, there is no direct anatomical demonstration of neural fibers from the central/peripheral nervous system penetrating into the gut.^24,78^ Therefore, further knowledge on the neurological cross talk with the gut and its microbiome is needed.^79^

## Supplementary Material





## Notes

Gut bacteria abundance across treatments (XLSX)
Additional information on experimental methods, chemical stability results, statistical results, biplots of bacteria abundance vs selected responses, enriched KEGG, and MetaCyc pathways (PDF)
