# Supplementary material for: From Gut to Brain: Glyphosate and Triclosan Impair Microbiome Composition, Neuroactive Metabolites, and Cognitive and Ecological Fitness in Daphnia magna
Source: Environ Sci Technol. 2026 Jan 5;60(2):1733–44. doi: 10.1021/acs.est.5c15302 (PMC12825155; doi:10.1021/acs.est.5c15302)
Supplement: Supplementary file 2 [file es5c15302_si_002.pdf]

Supplementary Information

From gut to brain: glyphosate and triclosan impair microbiome composition, neuroactive metabolites, and cognitive and ecological fitness in *Daphnia magna.*

Irene Romero-Alfano [a,b], Alba Julia López [a] , Benjamin Piña [a], Cristian Gómez-Canel [b], Carlos Barata [a*].

a Instituto de Diagnostico Ambiental y Estudios del Agua (IDAEA-CSIC), Department of Environmental Chemistry, Jordi Girona 18, Barcelona, 08034, Spain
b Universitat Ramon Llull IQS School of Engineering, Department of Analytical and Applied Chemistry, Via Augusta 390, Barcelona,08017, Spain

**corresponding author*

Number of pages 15, Tables 5 , Figures 5

Contents

Abbreviations	S3
Material and methods	S3
Experimental design (Fig S1)	S3
Chemical analysis	S4
Behavioral responses	S4
DNA extraction	S5
16S rRNA gene sequencing	S5
Metabolome analysis	S6
Recovery values for neurotransmitter and related metabolite extraction from brain and gut. (Fig S2)	S7
Results	S8
Heatmap of the enriched KEGG pathways (Fig S3)	S8
Scatterplots of the mean bacteria abundance vs selected relative concentration/responses of head and gut metabolites upon glyphosate exposure (Fig S4)	S9
Scatterplots of the mean bacteria abundance vs selected relative concentration/responses of head and gut metabolites upon triclosan exposure (Fig S5)	S10
Tables	S11
Table S1. Stability of glyphosate and triclosan across 48 hours.	S11
Table S2. Statistical results of behavioural and reproduction responses across glyphosate and triclosan concentrations and experiments	S12
Table S3. Adult body size results across experiments upon exposure to glyphosate and triclosan	S13
Table S4. PERmutational Multivariate ANOVA (PERMANOVA) results	S14
Table S5. Enriched MetaCyc pathways across triclosan (TCS) and glyphosate (Gly) treatments of gut microbiomes	S15
References	S17

Abbreviations

Metabolites

5-HIAA, 5-Hydroxyindoleacetic Acid; 5-HT, serotonin; 5-HTP, 5-Hydroxytryptophan, 3-MT, 3-Methoxytyramine; Ach, acetylcholine; Cho, choline; DA, dopamine; Epi, epinephrine; GABA, γ-Aminobutyric acid; Glu, L-Glutamic acid; L-DOPA, Levodopa; Oct, octopamine; Phe, phenylalanine; NE, norepinephrine; Tym, tyramine; Tyr, tyrosine; Tryp, tryptophan.

Life-history

N, total offspring production, r, intrinsic rate of population increase; age, age at first reproduction, egg, offspring size.

Behaviour

Basal, basal locomotion activity under darkness; VMR, visual motor response relative to basal activity during the first 5 min of light ; Max, maximal response within the first 10 light stimuli; H, habituation to repetitive light stimuli.

MATERIALS AND METHODS

Experimental design

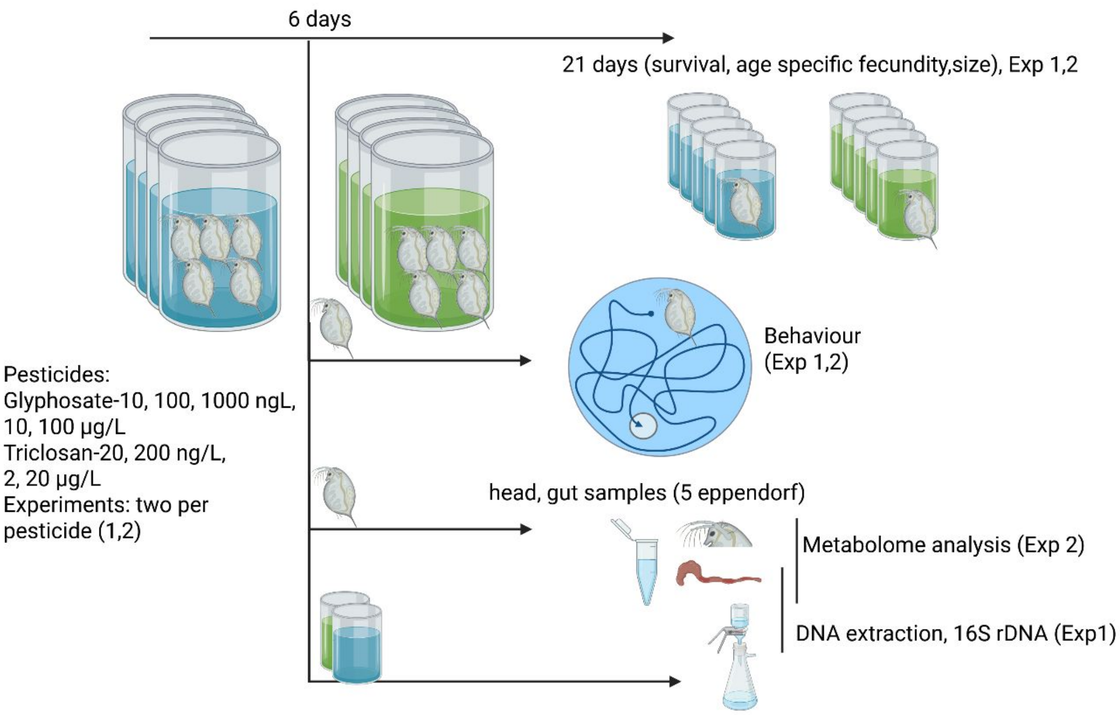

Figure S1. Schematic representation of experimental design.

Chemical analysis

Stability studies were carried out by analysing the compounds using LC-MS/MS (QTrap 7500, SCIEX). To improve the retention of glyphosate in the chromatography system, it was necessary to perform a derivatization step using Fmoc reagent, while triclosan was analysed by direct injection. In both cases, samples were filtered prior to injection. Once the sample preparation procedure was established, the LC-MS/MS method optimization was performed.

Ultra-pure Milli-Q water and acetonitrile HPLC-MS/MS grade were acidified to 0.2% of formic acid and used as aqueous and organic mobile phases, respectively. In case of Fmoc-Glyphosate a gradient starting at 5% B and a constant flow of 0.3 mL/min was used, while for triclosan direct injection a gradient starting at 65% B and a constant flow of 0.3 mL/min was employed. Chromatographic separations were achieved with a Kinetex Polar C18 column (2.6 μm, 150 × 2.1 mm) and a Polar C18 guard column (2.6 μm, 2.1 mm ID), both purchased from Phenomenex (California, USA). Both gradients lasted 6 minutes and injected 10 μL of the sample.

Regarding the MS/MS method, both compounds used a Multiple Reaction Monitoring mode (MRM), and the analysis were performed were using the electrospray ionization source in the negative mode (ESI-). For Fmoc-Glyphosate, the precursor ion optimised was 390.10 and its product ions were 168.1 and 150.0 (quantifier and qualifier, respectively). Regarding triclosan, the selected precursor ion was 287.2 and the product ions were 218.9 and 34.9 (quantifier and qualifier, respectively).

For the quantification of the stability samples, external calibration curves were used.

Behavioral responses

Two tests were performed to study the effect of glyphosate and triclosan in *D. magna* basal, visual motor, maximal and habituation responses to light stimuli, using a DanioVision Observation Chamber (DVOC-0040) and following previous procedures [1–3].

Further information is in SI. Behavioural tests are based on the exposure to light stimuli, which entails a stress for the studied *D. magna* clone promoting anti-predatory scape responses such as increasing locomotion/swimming activity. Before the tests, individuals were kept in darkness for acclimatization during 20 min and trials were performed in 24 well plates, with one individual per well, at 20ºC. The first test was based on monitoring basal locomotion activity during 5 min of darkness and visual motor responses upon the exposure to light during 5 minutes. In the second test individuals were exposed to 30 consecutive light flashes (1 second-light followed by 4 seconds of dark) to assess non associative learning responses such as the maximal response to first stimuli and habituation to repetitive light stimuli. Both tests were conducted consecutive with 24 biological replicates per condition randomized across plates and treatments. During all the tests, videos were recorded and responses were measured as distance moved.

DNA extraction

DNA extraction of gut *D. magna* samples followed the phenol-chloroform method described before [4] with few modifications. These included a previous step of three freeze and thaw cycles and homogenization with DNA lysis buffer using a TissueLyser® (Qiagen, Germantown, MA, USA). Total DNA was re-suspended in 50 µl of RNAse free water. DNA extraction of each filter was performed employing the kit DNeasy PowerSoil Pro Kit (Qiagen, Hilden, Germany) and total DNA was eluted in 50 µl of kit elution buffer (10 mM Tris-HCl pH 8.5). For each water sample, DNA extracts obtained from 3µm and 0.2µm membrane filters were pooled for latter analyses. The quality and quantity of total DNA was determined in a NanoDrop Spectrophotometer 8000 (Thermo Fisher Scientific, Inc). Extracted DNA samples were stored at -20 ◦C.

16S rRNA gene sequencing

Total DNA preparations were 16S gene fragments of rRNA gene were amplified using bar-coded universal primers directed to the bacterial V3-V4 16s rRNA region (341F

CCTAYGGGRBGCASCAG, 806R GGACTACNNGGGTATCTAAT) (Novogene Europe, UK). All PCR reactions were carried out with Phusion® High-Fidelity PCR Master Mix (New England Biolabs). Quality-checked PCR products were mixed at equal density ratios and purified by Qiagen Gel Extraction Kit (Qiagen, Germany). Sequencing libraries were generated using NEBNext Ultra DNA Library Pre ®Kit for Illumina, following manufacturer's recommendations, and index codes were added. Libraries were prepared and amplicons were sequenced on a paired-end Illumina platform generating 250 bp raw reads, and after reads merging, chimera removal and DADA2 denoising, ASVs (Amplicon Sequence Variables) were obtained. Annotation of ASVs was done using the SILVA database (v.138) through the Classify-sklearn moduler in QIIME2 software. ASV counts were rarefied using the package *vegan* (v. 2.6.4, Oksanen et al., 2022, software R, v. 4.2.1) prior to data analysis. Relative abundances were calculated, when necessary, as the abundance of a given taxa divided by the sum of all of them.

Metabolome analysis

For the neurotransmitters extraction and their metabolites from both the brain and the gut a previously optimized procedure [5] for the extraction of neurotransmitters in whole *Daphnia magna*, was used. The only difference was that the samples were reconstituted to a final volume of 50 µL instead of 100 µL due to the smaller amount of tissue in the collected samples. The above mentioned method provided good recoveries of most tested metabolite standards as it shown in Fig S2.

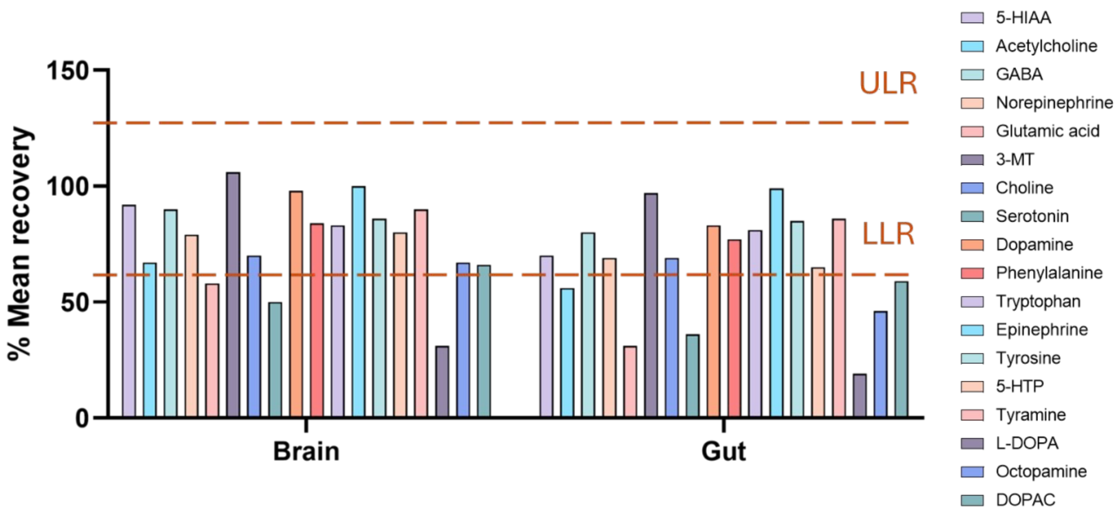

Figure S2. Recovery values for neurotransmitter and related metabolite extraction from brain and gut. LLR and ULR are, respectively, lower limit of recovery and upper limit of recovery.

Samples were analysed using liquid chromatography coupled to a triple quadrupole mass spectrometer (UHPLC-MS/MS). Specifically, the analysis method used had been previously optimised [6] on a Exion LC™ liquid chromatograph coupled to a Triple Quad 7500 System mass spectrometer (QTrap® Ready, SCIEX, USA).

The separation was based in the use of an Acquity UHPLC BEH Amide column (150 × 2.1 mm, 1.7 µm) with a matching pre-column (5 × 2.1 mm, 1.7 µm) (Waters, Milford, MA, USA). Aqueous solvent (A) consisted of Milli-Q water/acetonitrile (95:5) with 100 mM ammonium formate, while organic solvent (B) was composed of Milli-Q water/acetonitrile (15:85) with 30 mM ammonium formate, both adjusted to pH 3 with formic acid. A gradient of both mobile phases was set with a total analysis time of 10 minutes.

Regarding the detection system, a Multiple Reaction Monitoring (MRM) method was used to register simultaneously 17 compounds of interest, each one having its own precursor ion and two product ions. All the compounds were analysed using the electrospray ionization source in the positive mode (ESI+). Samples were quantified using calibration curves

constructed with internal standards, which were added prior to analysis to ensure accurate measurement.

RESULTS

KEGG pathways	Total	Hits-total	p	Hits relative to total GlyH	GlyG	TCNH	TCSG
Tyrosine Metabolism	70	8	<0.001	7	7	6	8
Catecholamine Biosynthesis	20	5	<0.002	4	4	4	5
Tryptophan Metabolism	59	5	0.002	5	5	1	3
Phenylalanine and Tyrosine Metabolis	27	3	0.008	2	3	1	3
Phospholipid Biosynthesis	29	3	0.045	2	3	1	2

% coverage

100 30

Figure S3. Heatmap of the enriched KEGG pathways of measured metabolites. GlyH, G; TCNH, G are respectively glyphosate and triclosan heats and guts, respectively

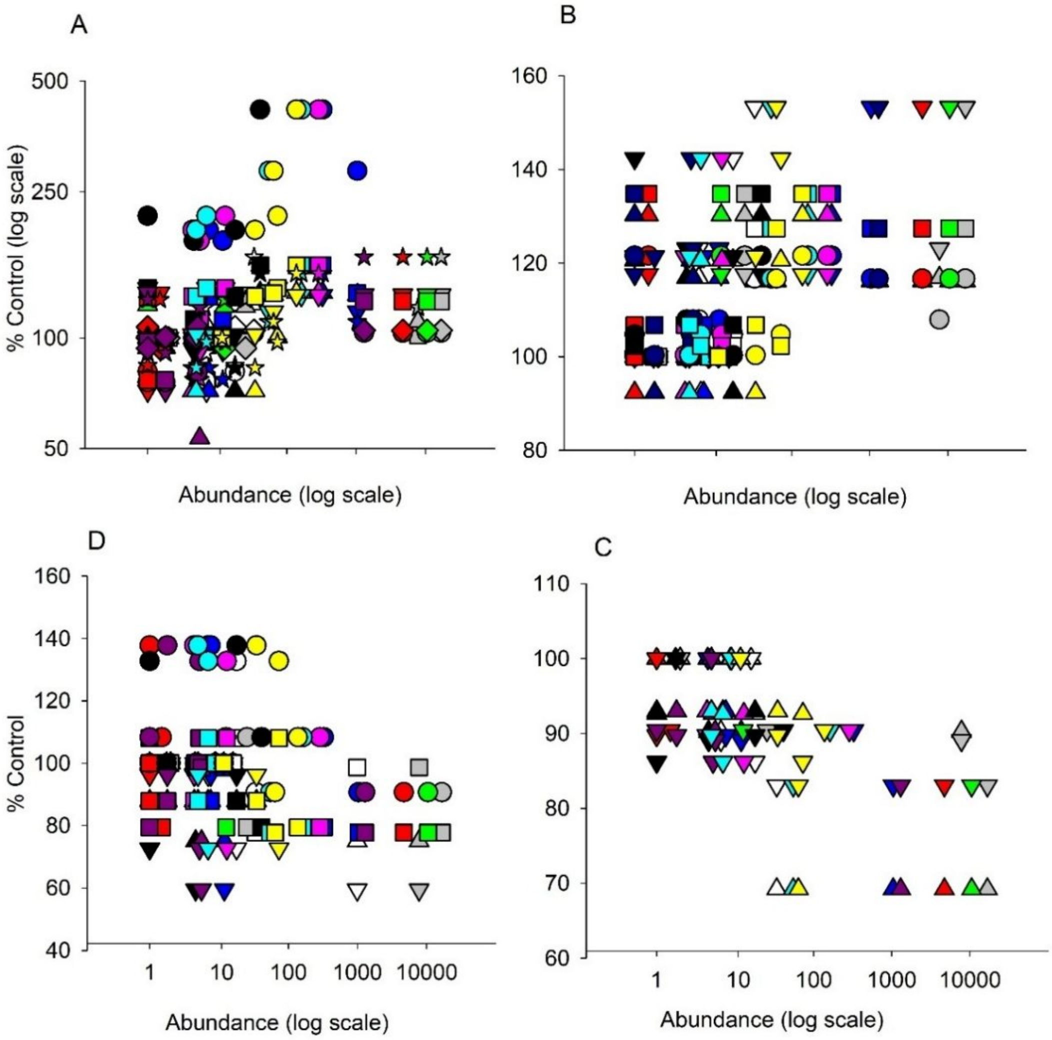

Figure S4. Scatterplots of the mean bacteria abundance vs selected relative concentration/responses of head and gut metabolites (A, C), anxiogenic behavioural (VMR, Max; B) and reproductive (N,r; D) traits from the first Glyphosate Cluster from Fig 4A. For clarity only the first ten ASVs are plotted in each graph. Different colours and symbol shapes represent, respectively, distinct ASVs and traits, respectively. Graphs A and B show positive correlations, whereas graphs C and D negative ones. Metabolites in graph A include H-Tyr, Oct, Epi, DA, GABA; G-5HTP, GABA, 5HT, 5HIAA; in graph C H-5HT, NE,3-MT; G-NE.

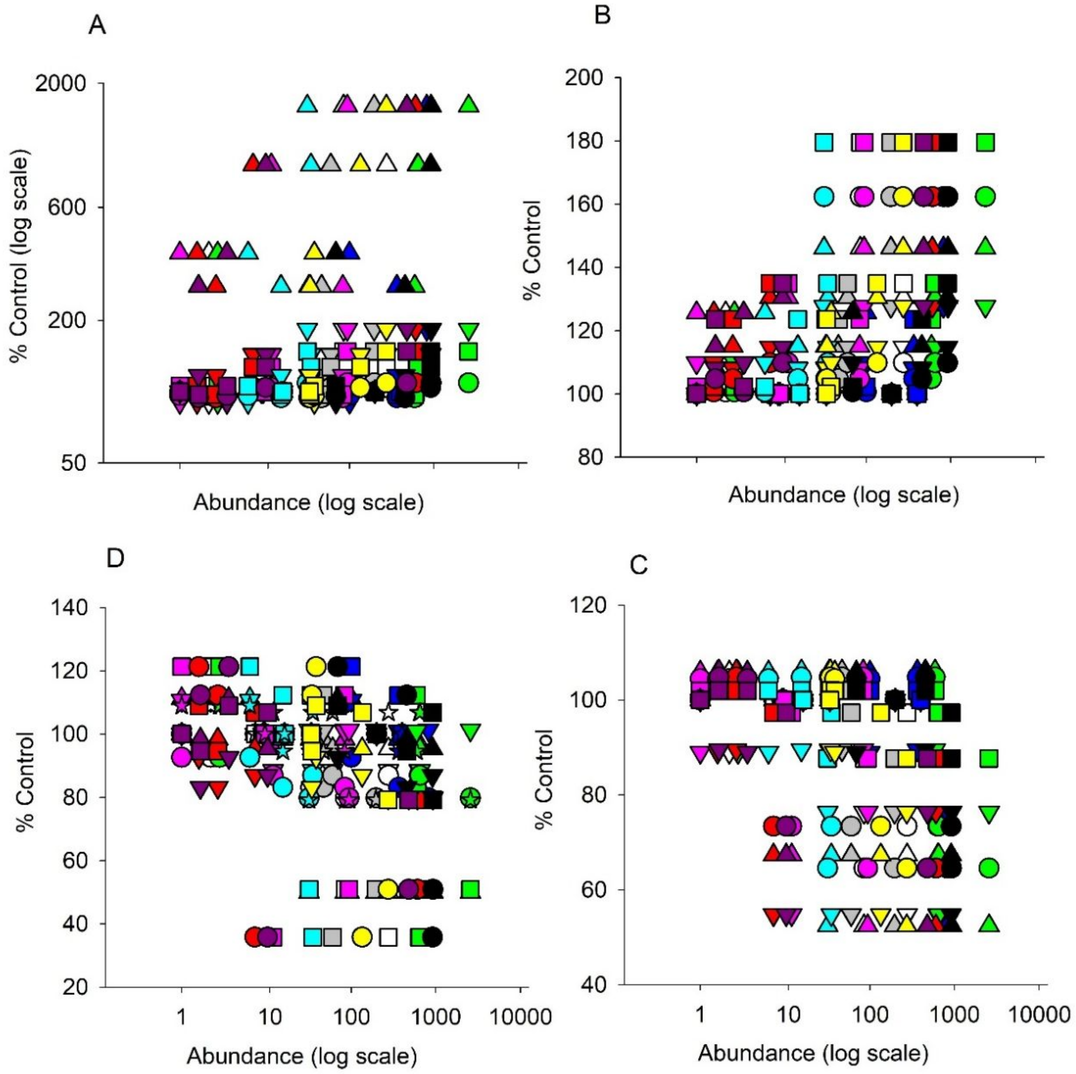

Figure S5. Scatterplots of the mean bacteria abundance vs selected relative concentration/responses of head and gut metabolites (A, C), anxiogenic behavioural (VMR, Max-B) and reproductive (N,r-D) traits from the first Triclosan Cluster from Fig 4B. For clarity only the first ten ASVs are plotted in each graph. Different colours and symbol shapes represent, respectively, distinct ASVs and traits, respectively. Graphs A and B show positive correlations, whereas graphs C and D negative ones. Metabolites in graph A include H-5HT, DA, GABA; G-5H,T in graph C H-Epi, 5HIAA; G-3MT,GABA, 5HTP

Tables

Table S1. Stability of glyphosate and triclosan across 48 hours. Results are expressed as Mean ± SD (N=4), in µg/L.

	Nominal	0 h	24 h	48 h
Glyphosate	10	10.3 ± 0.1	9.4 ± 0.2	9.1 ± 0.4
	100	101.0 ± 0.8	96.5 ± 0.4	98.7 ± 1.1
Triclosan	2	2.1 ± 0.1	1.8 ± 0.1	1.7 ± 0.2
	20	20.0 ± 0.2	19.3 ± 0.2	18.1 ± 0.3

Table S2. Statistical results of two way ANOVAs of behavioural and reproduction responses across glyphosate and triclosan concentrations and experiments. [1]Offspring length was compared by one way ANOVA. [2]Age at first reproduction values in both experiments were compared by separated non parametric Kruskal-Wallis test. df, F, P are, respectively degrees of freedom, Fisher's coefficient and probability. [2]Chi-square instead of F was used.

	Experiment			Treatment			Interaction		
Glyphosate	df	F	P	df	F	P	df	F	P
Behaviour									
Max	1,262	47.7	<.001	5,262	7.4	<.001	5,262	1.4	0.223
H	1,265	60.9	<.001	5,265	0.7	0.619	5,265	2.5	0.029
Basal	1,266	14.6	<.001	5,266	2.8	0.017	5,266	6.1	<.001
VMR	1,244	3.1	0.08	5,244	5.3	<.001	5,244	2.7	0.022
Reproduction									
Offspring	1,98	9.1	0.003	5,98	3.8	0.003	5,98	1.2	0.323
Body length	1,104	92.2	<.001	5,104	0.8	0.526	5,104	1.6	0.179
r	1,98	24.8	<.001	5,98	4.8	<.001	5,98	0.7	0.59
offspring length[1]				5,74	2.5	0.041			
Age_Experiment 1[2]				5	15.2	0.009			
Age_Experiment 2[2]				5	19	0.002			
Triclosan									
Behaviour									
Max	1,223	1.2	0.283	4,223	5.2	<.001	4,223	0.5	0.768
H	1,221	0.2	0.657	4,221	4.4	0.002	4,221	0.2	0.946
Basal	1,224	5.2	0.024	4,224	4.4	0.002	4,224	0.7	0.624
VMR	1,201	0.3	0.557	4,201	8.6	<.001	4,201	0.5	0.721
Reproduction									
Offspring	1,85	116.9	<.001	4,85	19.4	<.001	4,85	1.6	0.194
Body length	1,84	141.5	<.001	4,84	2.1	0.086	4,84	2.4	0.059
r	1,85	78.5	<.001	4,85	22.9	<.001	4,85	9.8	<.001
offspring length[1]				4,72	6	<.001			
Age_Experiment 1[2]				4	18.5	<.001			
Age_Experiment 2[2]				4	0.9	0.915			

Table S3. Adult body size results across experiments upon exposure to glyphosate and triclosan

	Exp1		Exp2	
Glyphosate	Mean	SE	Mean	SE
Control	3.85	0.04	3.56	0.04
10 ng/L	3.8	0.05	3.66	0.06
100 ng/L	3.84	0.06	3.64	0.06
1 µg/L	3.86	0.05	3.54	0.06
10 µg/L	3.97	0.04	3.61	0.04
100 µg/L	3.94	0.05	3.58	0.03
Triclosan	Mean	SE	Mean	SE
Control	3.85	0.03	3.58	0.03
20 ng/L	3.92	0.02	3.71	0.05
200 ng/L	3.94	0.02	3.63	0.06
2 µg/L	3.98	0.04	3.61	0.04
20 µg/L	3.95	0.04	3.51	0.08

Table S4. PERmutational Multivariate ANOVA (PERMANOVA) results analysing differences among water and gut bacterial communities by PPP (glyphosate and triclosan), concentration (low ≤ 1 µg/L and high>1 µg/L) and their interaction. Glyphosate and triclosan concentrations have been either grouped as low ≤ 1 µg/L and high>1 µg/L and compared in design 1; or ungrouped and tested separately in design 2. Further details are explained in methods.

	**df**	**R^2**	**F**	**P**
Water[1]				
PPP	1,21	0.23	9.3	0.001
Concentration	1,21	0.13	5.3	0.001
Interaction	1,21	0.12	4.7	0.001
Gut[1]				
PPP	1,50	0.13	8.7	0.001
Concentration	1,50	0.11	7.5	0.001
Interaction	1,50	0.04	2.6	0.02
Gut[2]				
Glyphosate	1,24	0.13	3.6	0.006
Triclosan	1,26	0.17	5.4	0.001

Table S5. Enriched MetaCyc pathways across triclosan (TCS) and glyphosate (Gly) treatments of gut microbiomes. % of bacteria in cluster 1 in Fig 4 belonging to them has also been included. Red and blue are respectively , up and down regulated. Values mean fold change in high vs low exposure levels *0.01<P<0.05; **0.001<P<0.01; *** P<0.001. White cells are no significant (P>0.05)

		Fold change		%	
Pathway (codes)	Names	TCS	Gly	TCS	Gly
PWY-6581	Spirilloxanthin biosyn.	437.1***	1.9*	18.8	17.6
P23-PWY	Reductive TCA cycle I	65.3***	2.5*		
3-HYDROXYPHENYLACETATE-DEGRADATION-PWY		41.9***	1.9*	31.3	23.5
P124-PWY	Bifidobacterium shunt	25***	2.9**	6.3	
P281-PWY	3-Phenylpropanoate deg.	21.3***	1.8*	25	17.6
GALLATE-DEGRADATION-I-PWY		8.6**	1.9*	25	17.6
P184-PWY	protocatechuate deg. I	5.1**	1.7*		
ANAEROFRUCAT-PWY		2.5**	1.5*	12.5	11.8
PWY-5676	Acetyl-CoA fermentation	1.8	2.1*		
SALVADEHYPOX-PWY	Aenosine nucleotides deg. II	2.9**	1.2	50	
PWY-1361	Benzoyl-CoA deg. I	10.4***	0.6		
GLUCUROCAT-PWY	β-D-glucuronides deg.	6.3**	1.6		
PWY-621	Sucrose deg. III	4.2**	1.2	50	
PWY-5384	Sucrose deg. IV	3.9**	1.3	31.3	
POLYAMSYN-PWY		3.9**	1.6		
GLUCOSE1PMETAB-PWY		3.4**	1.2	31.3	
REDCITCYC	TCA cycle VI (Helicobacter)	1	2.1**	37.5	17.6
PWY-6263	Menaquinol-8 biosyn. II	0.7	2.5**		
PWY1G-0	Mycothiol biosyn.	10.2	0.1**		
P122-PWY	Heterolactic fermentation	5.9***	0.6*		
PWY-5531	3,8-divinyl-chlorophyllide a biosyn. II	4.3***	0.1***		
PWY-7159	3,8-divinyl-chlorophyllide a biosyn. III	4.3***	0.1***		
KETOGLUCONMET-PWY	KETOGLUCONMET-PWY	3.9**	0.4*	6.3	
PWY-7013	(S)-Propane-1,2-diol deg.	27.3	0.2**		
PWY-5005	Biotin biosyn. II	16.6	0.1**		
PWY-5507	Adenosylcobalamin biosyn. I	2.7	0.1**		
PWY-6588	Pyruvate fermentation	2.5	0.1*		
PWY-6876	Isopropanol biosyn.	2.5	0.1*		
ALL-CHORISMATE-PWY	ALL-CHORISMATE-PWY	2.2	<0.1**		
PWY-7616	Methanol oxidation to CO2	2.1	0.1**		
PWY-6728	Methylaspartate cycle	2	0.1**		
PWY-5088	L-glutamate deg. VIII	1.6	0.1**		
P381-PWY	Adenosylcobalamin biosyn. II	1.3	0.1**		
TEICHOICACID-PWY	Poly(glycerol phosphate) acid biosyn.	1	0.1**		

PWY-5420	Catechol deg. II	0.7	0.1**		
PWY-6338	Vanillin and vanillate deg.	0.6**	0.2		
PWY0-1338	Polymyxin resistance	0.6**	0.1	12.5	
PWY-6071	Phenylethylamine deg.	0.5**	0.6		
DHGLUCONATE-PYR-CAT-PWY	DHGLUCONATE-PYR-CAT-PWY	0.4**	0.6	12.5	
PWY-5417	Catechol deg. III	0.4**	0.6	25	
PWY-5431	Aromatic compounds deg.	0.4**	0.6	25	
PWY-6185	4-Methylcatechol deg.	0.3**	0.5		
PWY-6505	L-Tryptophan deg. XII	0.3**	<0.1		
PWY-6210	2-Aminophenol deg.	0.3**	<0.2		
PWY-4722	Creatinine deg. II	0.3**	1.8		
POLYAMINSYN3-PWY	POLYAMINSYN3-PWY	0.3***	0.5		
DENITRIFICATION-PWY	DENITRIFICATION-PWY	0.3**	1.1		
NAD-BIOSYNTHESIS-II	NAD-BIOSYNTHESIS-II	0.2***	0.4	6.3	
PWY-6507	4-deoxy-L-threo-hex-4-enopyranuronate deg.	0.2**	0.4	12.5	
PWY0-1261	Peptidoglycan recycling I	0.4**	0.6	6.3	
PWY0-1296	Purine ribonucleosides deg.	0.3***	0.1	6.3	
PWY-7237	Myo-, chiro- and scyllo-inositol deg.	0.6**	0.2*	12.5	17.6
PWY-5920	Heme b biosyn. from glycine	0.5**	0.1***		
PWY0-1415	Heme b biosyn. from uroporphyrinogen-III	0.5**	0.1**		
P562-PWY	Myo-inositol deg. I	0.5**	0.2*	12.5	17.6
PWY-5838,5840,5897-99	Menaquinol biosyn.	0.5**	0.1**		
PWY-5861	Demethylmenaquinol-8 biosyn. I	0.5**	0.1**		
PWY-6396	2,3-butanediol biosyn.	0.5**	0.2***	6.3	
PWY-5863	Phylloquinol biosyn.	0.5**	0.1**		
PWY-5837	2-carboxy-1,4-naphthoquinol biosyn.	0.5**	0.1**		
PWY-6545	Pyrimidine deoxyribonucleotides de novo biosyn. III	0.5**	0.1***		
PWY-6383	Trans,octa-cis decaprenyl phosphate biosyn.	0.4**	0.1***		
PWY-7007	Methyl ketone biosyn.	0.4***	0.3**		
P125-PWY	(R,R)-butanediol biosyn.	0.4**	0.1**	12.5	
CATECHOL-ORTHO-CLEAVAGE-PWY	CATECHOL-ORTHO-CLEAVAGE-PWY	0.4**	0.5*	25	11.8
PWY0-845	Pyridoxal 5'-phosphate biosyn. and salvage	0.4**	0.1*	18.8	11.8
PRPP-PWY	Histidine, purine, and pyrimidine biosyn.	0.4**	0.2*	6.3	17.6
PROTOCATECHUATE-ORTHO-CLEAVAGE-PWY	PROTOCATECHUATE-ORTHO-CLEAVAGE-PWY	0.4**	0.5*	25	5.9

FERMENTATION-PWY	Mixed acid fermentation	0.4**	0.2***	37.5	29.4
PWY-6901	Glucose and xylose deg.	0.3***	0.4***		
PWY0-1298	Pyrimidine deoxyribonucleosides deg.	0.3**	0.1*		
P161-PWY	Acetylene deg.	0.3**	0.1***	6.3	5.9
P221-PWY	Octane oxidation	0.3**	0.3*	18.8	5.9
ARGORNPROST-PWY	L-arginine deg. VI	0.3**	0.2*	18.8	5.9
PWY0-1297	Purine deoxyribonucleosides deg.	0.3**	0.1*	6.3	17.6
BIOTIN-BIOSYNTHESIS-PWY	BIOTIN-biosyn.-PWY	0.3***	0.2**	37.5	23.5
PWY-7376	Cob(II)yrinate a,c-diamide biosyn. II	0.3**	0.5*	18.8	5.9
PWY-6519	8-amino-7-oxononanoate biosyn. I	0.3***	0.2**	37.5	23.5
PWY-5415	Catechol deg. I	0.3***	0.1**		
PWYG-321	Mycolate biosyn.	0.3***	0.1**	6.3	
PYRIDOXSYN-PWY	PYRIDOXSYN-PWY	0.3***	0.2**	25	23.5
PWY-7664	Oleate biosyn. IV	0.3***	0.1**	37.5	23.5
PWY-5989	Stearate biosyn. II (bacteria and plants)	0.3***	0.1**	37.5	23.5
PWY0-862	(5Z)-dodecenoate biosyn. I	0.3***	0.1**	37.5	23.5
PWY-6282	Palmitoleate biosyn. I	0.3***	0.1**	37.5	23.5
FASYN-INITIAL-PWY	FASYN-INITIAL-PWY	0.3***	0.1**	37.5	23.5
PWY0-1533	Methylphosphonate deg. I	0.2***	0.3**		5.9

References

(1) Bedrossiantz, J.; Martínez-Jerónimo, F.; Bellot, M.; Raldua, D.; Gómez-Canela, C.; Barata, C. A High-Throughput Assay for Screening Environmental Pollutants and Drugs Impairing Predator Avoidance in Daphnia Magna. *Science of the Total Environment* 2020, *740*. https://doi.org/10.1016/j.scitotenv.2020.140045.

(2) Bellot, M.; Gómez-Canela, C.; Barata, C. Phototactic Behaviour and Neurotransmitter Profiles in Two Daphnia Magna Clones: Vertical and Horizontal Responses to Fish Kairomones and Psychotropic Drugs. *Science of the Total Environment* 2022, *830*. https://doi.org/10.1016/j.scitotenv.2022.154684.

(3) Bellot, M.; Faria, M.; Gómez-Canela, C.; Raldúa, D.; Barata, C. Pharmacological Modulation of Behaviour, Serotonin and Dopamine Levels in Daphnia Magna Exposed to the Monoamine Oxidase Inhibitor Deprenyl. *Toxics* 2021, *9* (8). https://doi.org/10.3390/toxics9080187.

(4) Fuertes, I.; Barata, C. Characterization of Neurotransmitters and Related Metabolites in Daphnia Magna Juveniles Deficient in Serotonin and Exposed to Neuroactive Chemicals That Affect Its Behavior: A Targeted LC-MS/MS Method. *Chemosphere* 2021, *263*, 127814. https://doi.org/10.1016/j.chemosphere.2020.127814.

(5) Romero-Alfano, I.; Stevanović, M.; Goyenechea, J.; Prats, E.; Barata, C.; Raldúa, D.; Gómez-Canela, C. Ultra-Sensitive UHPLC-MS/MS Method for Simultaneous Quantification of 31 Neurochemicals in Zebrafish Larvae and Brain. *Talanta* 2025, *295*, 128334. https://doi.org/10.1016/j.talanta.2025.128334.
